# *In silico* design of a novel multi-epitope mRNA vaccine candidate for BtHKU5-CoV-2 using immunoinformatics

**DOI:** 10.1371/journal.pntd.0013517

**Published:** 2026-04-03

**Authors:** Ningze Zheng, Yingqi Xu

**Affiliations:** 1 The First Affiliated Hospital of Guangzhou Medical University, Guangzhou, China; 2 EnCureGen Pharma, Guangzhou, China; 3 Department of Pharmacology, Zhongshan School of Medicine, Sun Yat-sen University, Guangzhou, China; The University of Kansas, UNITED STATES OF AMERICA

## Abstract

Bat HKU5-CoV-2 (BtHKU5-CoV-2), a recently discovered bat-infecting merbecovirus, was found to infect human cell lines by utilizing the human angiotensin-converting enzyme 2 (ACE2) receptor, similar to SARS-CoV-2, which caused millions of deaths. Moreover, its broad host tropism has raised significant concerns about potential human spillover risk. Therefore, there is an urgent need to develop vaccines to combat the potential outbreak of BtHKU5-CoV-2. However, research focusing on BtHKU5-CoV-2 remains limited. In this study, we designed a novel multi-epitope vaccine for BtHKU5-CoV-2 using an immunoinformatics approach. Eight cytotoxic T lymphocyte (CTL) epitopes, seven helper T lymphocyte (HTL) epitopes, and five linear B lymphocyte (LBL) epitopes were screened from the spike glycoprotein of BtHKU5-CoV-2. The selected epitopes were joined together with appropriate linkers, and β-defensin II and MHC I-targeting domain (MITD) were incorporated into the construct to enhance vaccine immunogenicity. *In silico* analyses suggested that the designed vaccine may have favorable predicted antigenicity and immunogenicity while being non-toxic and non-allergenic. The tertiary structure of the multi-epitope vaccine was modeled and refined, and its structural plausibility was evaluated using *in silico* quality metrics. Molecular docking studies suggested plausible interaction modes between the vaccine construct and Toll-like receptor 2 (TLR2) and TLR4. Moreover, the mRNA was predicted to show potential interaction modes with TLR3, TLR7, and TLR8 receptors. Additionally, *in silico* immune simulations suggested that vaccination may elicit humoral and cellular immune responses. Collectively, these computational results suggest that the proposed mRNA vaccine is a potential candidate for BtHKU5-CoV-2. However, further experiments are necessary to validate its protective efficacy.

## Author summary

BtHKU5-CoV-2, a newly discovered merbecovirus isolated from bats, exhibits potential for spillover into humans. It was found to utilize human ACE2 as a functional receptor for infection. The viral spike acts like a “key” that fits into the “lock” of the ACE2 receptor on the host cell, enabling viral entry. BtHKU5-CoV-2 warrants significant attention, because it shares the same functional receptor with SARS-CoV-1 and SARS-CoV-2, which caused the 2003 SARS epidemic and the 2019 pandemic, respectively. Thus, developing vaccines to prevent potential global outbreaks of BtHKU5-CoV-2 is urgently needed. Theoretically, within the body’s immune surveillance system, proteins from BtHKU5-CoV-2 are processed via proteasomal degradation into short peptides. The peptides with immunogenicity bind to MHC molecules and are presented on the cell surface. These peptides, known as epitopes, can initiate immune response. In this study, we designed a multi-epitope mRNA vaccine for BtHKU5-CoV-2 using immunoinformatics methods. Epitopes were screened from the spike glycoprotein, a promising target of BtHKU5-CoV-2. *In silico* analyses suggested that the proposed vaccine may have a favorable predicted safety profile and may have the potential to induce humoral and cellular immune responses. These computational findings warrant further experimental validation to assess its possible contribution to outbreak preparedness.

## Introduction

BtHKU5-CoV-2, a positive-strand RNA virus, was first reported in *Pipistrellus* bat anal swab samples collected from several provinces of China in 2025 [1]. It is a new merbecovirus belonging to the same family as other coronaviruses that infect humans, such as MERS-CoV. Like other coronaviruses, BtHKU5-CoV-2 employs the spike glycoprotein to recognize receptors and mediate the fusion of viral and cellular membranes [2]. More importantly, it was found to infect human cell lines by utilizing angiotensin-converting enzyme 2 (ACE2) as a functional receptor [1]. ACE2 is a known receptor of SARS-CoV-2, a zoonotic virus that has led to COVID-19 and caused millions of deaths in recent years [3,4]. Additionally, BtHKU5-CoV-2 may infect various mammalian and avian species via ACE2 orthologs [1]. Therefore, BtHKU5-CoV-2 has the potential for zoonotic transmission and threatens public health, raising immediate concerns [5–7].

mRNA vaccines play crucial roles in reducing hospitalizations and deaths caused by SARS-CoV-2 during the COVID-19 pandemic [8]. Since then, mRNA vaccine technology has rapidly developed and has become a frontline measure for defense against infectious diseases [9]. Compared to traditional recombinant protein vaccines and DNA vaccines, mRNA vaccines exhibit multiple advantages, including: (i) they are not incorporated into the host’s genome; (ii) mRNA can be produced in an *in vitro* cell-free system without complex purification procedures; (iii) multiple antigens can be inserted into one mRNA sequence; (iv) unlike DNA vaccines, mRNA vaccines do not induce an immune response against components of the vector [10]. Overall, mRNA vaccines are fast, cheap, and flexible tools to defend against rapid outbreaks of infectious diseases.

Advancements in immunoinformatics approaches and the availability of immunological datasets have helped researchers screen target epitopes and design epitope-based vaccines more efficiently. In particular, multi-epitope vaccines have gained great attention for rapidly mutating pathogens. On the one hand, researchers can identify potent conserved epitopes from viral components in a short time. On the other hand, cytotoxic T lymphocyte (CTL) epitopes, helper T lymphocyte (HTL) epitopes, and B cell epitopes can be incorporated into a multi-epitope vaccine, resulting in simultaneous activation of cellular and humoral immune responses, which are necessary for defense against infectious pathogens [11]. Moreover, inclusion of adjuvants and components that assist in epitope presentation boosts immunogenicity and extends immune responses [12,13]. In recent years, some studies have successfully designed multi-epitope vaccines targeting different viruses and achieved the desired protective effects, such as mRNA vaccines against monkeypox virus [14] and HIV mRNA vaccines [15].

Some researchers have become aware of BtHKU5-CoV-2 because of its spillover potential. Therefore, it is necessary to develop an effective vaccine to combat rapid viral outbreaks. Combining mRNA platforms and epitope design is an ideal strategy for developing a multi-epitope mRNA vaccine against BtHKU5-CoV-2. In this study, we predicted the conserved CTL, HTL, and B cell epitopes from the spike glycoprotein of BtHKU5-CoV-2. We selected promising epitopes for the multi-epitope vaccine development. Immunogenicity, tertiary structure, interaction with immune receptors, and physicochemical properties of the vaccine were predicted in our study. Overall, this study aims to design a multi-epitope mRNA vaccine candidate for BtHKU5-CoV-2 using immunoinformatics approaches. We hypothesize that the proposed vaccine—designed from conserved epitopes predicted to have favorable antigenicity and immunogenicity with low allergenicity and toxicity—will be safe and capable of eliciting effective immune responses, as demonstrated through *in silico* evaluation.

## Methods

### 1. Retrieval of target protein sequences

The amino acid sequences of the spike glycoproteins of BtHKU5-CoV-2–441 (C_AAI84074.1), BtHKU5CoV-2–153 (C_AAI84049.1), BtHKU5-CoV-2–155 (C_AAI84054.1), BtHKU5-CoV-2–023 (C_AAI84059.1), BtHKU5-CoV-2–028 (C_AAI84064.1), and BtHKU5-CoV-2–381 (C_AAI84069.1) were obtained from GenBase. Among these bat HKU5-CoVs, the spike glycoproteins share 91.2%-99.2% similarity in amino acid sequences, and BtHKU5-CoV-2–441 showed the highest identity with the others [1]. We chose BtHKU5-CoV-2–441 (referred to as BtHKU5-CoV-2 in the following analysis) as the representative strain for further study.

### 2. Epitopes prediction

#### 2.1. CTL epitopes prediction.

To identify the CTL epitopes of the target protein, NetCTL 1.2 (https://services.healthtech.dtu.dk/services/NetCTL-1.2/) was employed. In this step, a threshold of 0.75 (default value) was applied, while C-terminal cleavage, TAP transport efficiency, and peptide-MHCI binding were also considered. Twelve supertypes, A1, A2, A3, A24, A26, B7, B8, B27, B39, B44, B58, and B62, were considered in the screening process [16]. Antigenicity prediction of epitopes was subsequently performed using Vaxijen 2.0 tool (https://ddg-pharmfac.net/vaxijen/VaxiJen/VaxiJen.html) with a threshold of 0.4 [17]. To screen for non-toxic and non-allergenic epitopes, AllerTOP 2.0 (http://www.ddg-pharmfac.net/AllerTOP) and ToxinPred 2.0 (https://webs.iiitd.edu.in/raghava/toxinpred2/) were employed to exclude epitopes predicted to be allergenic or toxic [18,19]. The immunogenicity of epitopes is critical for activating the immune response. Therefore, the epitopes were submitted to the Class I Immunogenicity server in the IEDB platform (http://tools.iedb.org/immunogenicity/) [20]. Epitopes with positive predicted immunogenicity scores (score exceeding 0) were included in this study. Finally, to predict which MHC I alleles the epitopes were likely to bind, the TepiTool in the IEDB platform (http://tools.iedb.org/tepitool/) was applied, and the IC50 of selected epitopes should be less than 500 nM [21]. All prediction servers and their associated parameters and threshold values used in this study were summarized in S1 Table.

#### 2.2. HTL epitopes prediction.

Prediction of HTL epitopes was conducted using the NetMHCIIpan - 4.0 server (https://services.healthtech.dtu.dk/services/NetMHCIIpan-4.0/), which uses Artificial Neural Networks (ANNs) to predict peptides that are capable of binding to indicated MHC II molecules [22]. In this screening process, HLA-DRB1 alleles (including 0101, 0301, 0401, 0404, 0701, 0802, 0901, 1101, 1302, 1501), HLA-DRB3_0101, DRB4_0101, DRB5_0101, HLA-DQA10501 (-DQB10201, -DQB10301), HLA-DQA10301-DQB10302, HLA-DQA10401-DQB10402, HLA-DQA10101-DQB10501, HLA-DQA10102-DQB10602, HLA-DPA10201-DPB10101, HLA-DPA10103 (-DPB10201, -DPB10401), HLA-DPA10301-DPB10402 and HLA-DPA10201 (-DPB10501, -DPB11401) were selected for prediction. Only epitopes with an IC50 < 500 nM were subjected to further analysis. According to the default classification criteria of NetMHCIIpan - 4.0, peptides with %Rank ≤ 2% are defined as strong binders, whereas those with 2% < %Rank ≤ 10% are considered weak binders. The antigenicity, allergenicity, and toxicity of epitopes were characterized using Vaxijen 2.0, Allertop 2.0, and ToxinPred 2.0, respectively. Epitopes with an antigenicity score over the threshold of 0.4, while are non-toxic and non-allergenic were retained. Finally, the epitopes’ potential ability to induce IL-2, IL-4, and IFN-γ was evaluated using IL2Pred (https://webs.iiitd.edu.in/raghava/il2pred/), IL4Pred (https://webs.iiitd.edu.in/raghava/il4pred/) and IFNepitope (https://webs.iiitd.edu.in/raghava/ifnepitope/) [23,24]. The epitopes predicted to induce IL-2, IL-4, and IFN-γ were included in the following study.

#### 2.3. Linear B cell epitopes prediction.

For predicting linear B cell epitopes, we employed the ABCpred tool (https://webs.iiitd.edu.in/raghava/abcpred/) in this study [25]. In this step, the default settings were used, with a threshold of 0.51 and an epitope length of 16 amino acids for screening. Subsequently, antigenicity, allergenicity, and toxicity of the epitopes were assessed using Vaxijen2.0, Allertop 2.0, and ToxinPred 2.0, respectively. Epitopes that were predicted to have the characteristics of an antigenicity score higher than 0.4, non-toxic, and non-allergenic, were chosen for vaccine construction.

### 3. Epitope conservation analysis and homology study

The complete genomes of all six bat HKU5-CoV lineage 2 (HKU5-CoV-2) strains were downloaded from GenBase following accession numbers: C_AA085189.1, C_AA085190.1, C_AA085191.1, C_AA085192.1, C_AA085193.1, and C_AA085194.1. The nucleotide sequence of the spike glycoprotein was extracted from the corresponding genomic sequences. Conservation analysis of epitopes across the six bat HKU5-CoV-2 strains was conducted using the local *tblastn* tool. Epitopes that were highly conserved or lacked mutation sites were considered in the following study. To avoid cross-immunity or tolerance to epitopes, the complete human proteome and complete proteome of seventy-nine bacterial species that commonly reside in the human gut were downloaded from the NCBI database. A local database was established using Diamond software, and then the BLASTp program was employed to analyze the homologs between the candidate epitopes and human proteomes, as well as intestinal bacterial proteomes. The selected epitopes met the following criteria: E-value > 10^-4^ and bit score < 100.

### 4. Multi-epitope vaccine construction and physicochemical properties prediction

Following screening, epitopes with high predicted antigenicity and predicted non-toxicity and non-allergenicity were included in vaccine design. For joining the epitopes, AAY linkers were used for CTL epitopes, GPGPG linkers were used for HTL epitopes, while KK linkers were used for LBL epitopes. In addition, β-defensin II, an immunological adjuvant, was attached to the N-terminus of the sequences using the EAAAK linker. A TAT peptide, which is capable of assisting the vaccine in traversing the cell membrane, was added to the C- terminus of the vaccine and connected by an EAAAK linker. Finally, the ProtParam tool (https://web.expasy.org/protparam/), VaxiJen 2.0, ToxinPred2.0, and AllerTOP 2.0 were employed to predict the physicochemical properties, antigenicity, toxicity, and allergenicity of the vaccine, respectively.

### 5. Structure modeling of the vaccine

The secondary structure of the vaccine was predicted using PSIPRED 4.0 (https://bioinf.cs.ucl.ac.uk/psipred/), and elements such as strands, helices, and coils are shown in the structure. The tertiary structure of vaccine was modeled using the Robetta server (https://robetta.bakerlab.org/), which predicts the protein structure based on an accurate deep learning-based method RoseTTAFold [26]. Subsequently, the GalaxyRefine server (https://galaxy.seoklab.org/cgi-bin/submit.cgi?type=COMPLEX) was used for structural optimization of the model [27]. Only the refined model with a lower MolProbity score underwent quality assessment using ProSA-Web (https://prosa.services.came.sbg.ac.at/prosa.php), PROCHECK in the PDBsum platform, and ERRAT [28]. Visualization of the 3D structure of vaccine was performed using PyMOL v2.5 software.

### 6. Conformational B cell epitopes prediction

Spike glycoprotein-specific antibodies play a significant role in the prevention of coronavirus infection. The conformational B-cell epitopes contained in vaccine structures were predicted using ElliPro (http://tools.iedb.org/ellipro/), an antibody epitope prediction server [29]. The tool predicts discontinuous antibody epitopes according to the 3D shape of the protein and outputs a score (protrusion index, PI) for each predicted epitope.

### 7. Molecular docking

Two molecules were selected for complex docking: TLR2 and TLR4, which are important pattern recognition receptors in mammals. TLR2 and TLR4 can trigger innate antiviral responses via direct or indirect interactions with viral glycoproteins [30]. From the RCSB PDB database, the crystal structures of TLR2 and TLR4 were downloaded with PDB ID 2Z7X and 4G8A, respectively. After preprocessing, such as removing non-protein components, the tertiary structures were submitted to the ClusPro 2.0 server (https://cluspro.org/home.php) for molecular docking [31]. Subsequently, the structures of the complexes were refined using the HADDOCK 2.4 server (https://rascar.science.uu.nl/haddock2.4/), which drives the docking process based on information from predicted protein interfaces in ambiguous interaction restraints (AIRs). Finally, the predicted interactions within the complexes were analyzed using the PDBsum server (https://www.ebi.ac.uk/thornton-srv/databases/pdbsum/Generate.html).

### 8. Molecular dynamics simulation

Molecular dynamics (MD) simulations were conducted using the iMODs tool (https://imods.iqf.csic.es/) to evaluate the dynamic stability of the docking complex [32]. The online server explores the collective motion of proteins in internal coordinates using the normal modal analysis (NMA). Indicators, including flexibility and covariance, were used to assess the predicted stability of vaccine/TLR complexes.

### 9. Population coverage calculation and immune simulation analysis

Only in individuals who express specific HLA molecules that bind to and present corresponding epitopes on the cell surface can an epitope trigger an immune response. The expression and distribution of HLA subtypes vary in different countries and regions. Therefore, the Population Coverage server in the IEDB platform (http://tools.iedb.org/population/) was implemented to calculate the population coverage across the world for epitope sets contained in the vaccine [33]. In this program, the calculation options, class I and class II combined, were selected for analysis in the present study.

To predict the host’s immune response against vaccination, the C-IMMSIM webserver (https://kraken.iac.rm.cnr.it/C-IMMSIM/index.php?page=0) was employed to simulate the immune response profile following three vaccine injections [34]. HLA alleles, including HLA-A0201, HLA-A2301, HLA-B5301, HLA-B1501, HLA-DRB1_0405, and HLA-DRB1_0701, were selected for analysis. The duration of the simulation was 1000 time steps, which is approximately 350 days.

### 10. Construction of mRNA vaccine

In the final construction of the multi-epitope vaccine, the signal peptide of Tissue Plasminogen Activator, which enables the synthesized peptides to be secreted into the extracellular space, was added to the N-terminus of the sequence. Furthermore, the sequence of the MHC I-targeting domain (MITD) was attached to the C-terminus to improve the epitope presentation. The amino acid sequence of the vaccine was submitted to the GenSmart Codon Optimization tool of the GenScript platform for reverse translation and codon adaptation in *Homo sapiens*. Additionally, a Kozak sequence was added to the 5’ end, and a TAA codon was used to stop translation. To improve the stability and translation efficiency of the mRNA, a 5’-UTR (derived from cytomegalovirus immediate-early gene), 3’-UTR (derived from human growth hormone), and 120-nt polyadenylate tail were attached to the mRNA structure. Full-length DNA sequence was cloned into the pET-28a (+) vector using the GenSmart Design server on the GenScript platform. This vector was presumed to be linearized by BamHI restriction enzyme and used as a template for *in vitro* mRNA transcription.

### 11. mRNA-TLR molecules docking analysis

To predict the interactions between the mRNA of the vaccine and TLR molecules, the transcription tool was first utilized to transcribe the full DNA sequence, including the region from the 5’-UTR to the poly(A) tail, into mRNA. Subsequently, the RNAfold tool (http://rna.tbi.univie.ac.at/cgi-bin/RNAWebSuite/RNAfold.cgi) was used to predict the secondary structure of the vaccine mRNA.

Endosomal TLRs, including TLR3, TLR7, and TLR8, specialize in recognizing the nucleic acids of pathogens and subsequently activate the host’s innate immune system [35]. Therefore, potential interactions between TLRs and the mRNA vaccine sequence were assessed *in silico* in the present study. The amino acid sequences of human TLR3, TLR7, and TLR8 were obtained from the UniProt database with the accession numbers O15455, Q9NYK1, and Q9NR97, respectively. RNA-protein interactions were predicted using the RPISeq tool (http://pridb.gdcb.iastate.edu/RPISeq/batch-rna.html), which outputs the results in the RF and SVM classifier forms. Finally, the crystal structure of TLR molecules that predicted to interact with the mRNA vaccine was retrieved from the RCSB PDB database, and the molecular docking between TLR molecules and vaccine mRNA was performed using the HDOCK server.

## Results

### 1. Prediction of epitopes

Using several credible epitope prediction tools, we identified eight CTL epitopes (Table 1), seven HTL epitopes (Table 2), and five LBL epitopes (Table 3) in the spike glycoprotein sequence of the BtHKU5-CoV-2–441 strain. All selected epitopes were predicted to have favorable antigenicity and to be non-toxic and non-allergenic. Further *in silico* analyses suggested that the HTL epitopes have the predicted potential to induce IFN-γ, IL-2, and IL-4. Among the LBL epitopes with a high predicted score of antigenicity, only those that lie within the receptor-binding domain of spike glycoprotein were selected [2].

**Table 1:** CTL epitope screening results.

Epitope	Start position	HLA molecule	Rank%	IC50 (nM)	Antigenicity	Conservation
KTYSNITIA	62	HLA-A*30:01	0.089	20.42	1.0509	6/6
TYSNITIAM	63	HLA-A*24:02	0.55	411.35	1.2420	6/6
		HLA-A*23:01	0.857	453.21		
LPYFHNINY	279	HLA-B*35:01	0.015	5.3	1.0163	6/6
		HLA-B*53:01	0.112	96.24		
VQMTFVISV	547	HLA-A*02:06	0.009	2.59	1.1614	6/6
		HLA-A*02:01	0.034	5.03		
		HLA-A*02:03	0.141	7.64		
VSIPTNFSF	757	HLA-B*58:01	0.021	5.93	1.1587	6/6
		HLA-B*57:01	0.069	24.48		
		HLA-B*15:01	0.337	60.24		
		HLA-A*32:01	0.124	83.44		
		HLA-A*23:01	0.345	110.43		
		HLA-B*35:01	0.359	159.06		
		HLA-A*24:02	0.482	329.45		
IPTNFSFGL	759	HLA-B*53:01	0.126	112.83	1.8318	6/6
MEAAYTASL	925	HLA-B*40:01	0.008	5.69	0.6652	6/6
LASFAAIPF	946	HLA-B*35:01	0.029	8.76	0.8943	6/6

**Table 2:** HTL epitope screening results.

Epitope	Start position	HLA II molecule	Rank%	IC50 (nM)	Antige- nicity	Toxin	Allergic	IFN-γ	IL-2	IL-4	Conserv- ation
TTARIKKIYPAFVLG	134	DRB1_0101	1.72	70.79	0.6896	_	_	+	+	+	6/6
TIVISPTTTARIKKI	127	DRB1_0401	0.07	82.72	0.7988	_	_	+	+	+	6/6
YFHNINYYSVIPRSP	281	DRB1_1501	0.66	91.21	0.4245	_	_	+	+	+	6/6
ISPTTTARIKKIYPA	130	DRB1_0701	1.09	121.48	1.0021	_	_	+	+	+	6/6
WTAGLASFAAIPFAQ	942	DRB1_1501	2.22	174.42	0.6589	_	_	+	+	+	6/6
LYFFHVGYQPTALIN	1116	HLA-DPA10103 -DPB10201	3.7	316.83	1.0424	_	_	+	+	+	6/6
RYVAMDVKFENITTK	1190	DRB1_0405	3.58	449.56	1.5141	_	_	+	+	+	6/6

**Table 3:** LBL epitope screening results.

Epitope	Start position	Antigenicity	Conservation
FPLSMASYLRPGSTGP	441	1.1750	6/6
SMTERVQMTFVISVTF	542	1.1665	6/6
DVESGVYSVSSFEAKS	348	1.0387	6/6
PGSTGPTAEFNYRQDF	451	1.0319	6/6
SDGQSASMTERVQMTF	536	0.8603	6/6

By mean of the local *tblastn* tool, we analyzed the conservation of selected epitopes across the six BtHKU5-CoV-2 strains, and the results revealed that all epitopes showed evident conservation or complete identity. These findings suggested that the vaccine construct designed for the BtHKU5-CoV-2–441 strain may offer broader theoretical coverage across additional strains, but this warrants experimental validation.

### 2. Vaccine construction and physicochemical properties prediction

**Fig 1:**
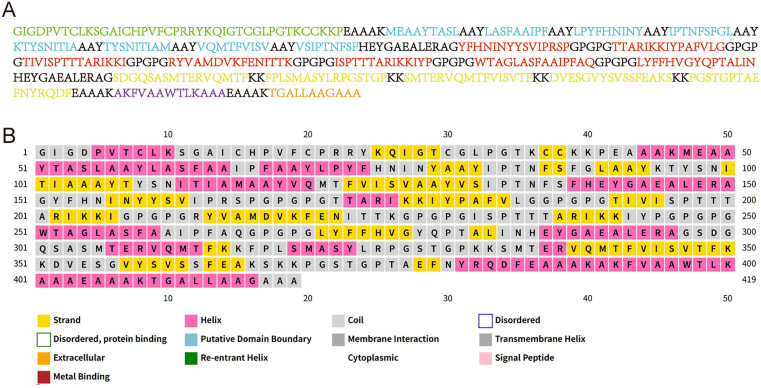
Schematic illustration of BtHKU5-CoV-2 vaccine construction. **(A)** The amino acid sequence of the vaccine construct. Each part is represented in a different color, with β-defensin II adjuvant, CTL epitope, HTL epitope, LBL epitope, pan-HTL epitope and TAT peptide shown in green, blue, red, yellow, purple and orange, respectively. **(B)** The predicted secondary structure of the vaccine construct. Distinct structural elements labeled with different colors are indicated in the annotations below.

In the vaccine design process, CTL epitopes, HTL epitopes, and LBL epitopes are joined by AAY, GPGPG, and KK linkers, respectively. Furthermore, β-defensin II was attached to the N-terminus, whereas a TAT peptide was added to the C-terminus (Fig 1A). The vaccine is composed of 419 amino acids and has a molecular weight of 44.37 kDa. The antigenicity of the vaccine was predicted by VaxiJen 2.0 and ANTIGENpro servers, and the scores were 0.7537 and 0.943474, suggesting that the vaccine may have considerable antigenic potential. Meanwhile, analyses using ToxinPred2.0 and AllerTOP 2.0 predicted no allergenicity or toxicity for the vaccine construct. Using the ProtParam program, it was predicted that the vaccine’s theoretical isoelectric point value was 9.56, its instability index was 25.81, and its aliphatic index was 70.31, suggesting that the vaccine protein has the potential to be stable. The half-life of the vaccine was estimated *in silico* to be 30 h in mammalian reticulocytes (*in vitro*), > 20 h in yeast, and >10 h in *Escherichia coli*. In addition, the solubility value was calculated to be 0.515 using the Protein-Sol web tool, implying that the vaccine has high predicted solubility, is unlikely to aggregate, and is suitable for *in vivo* expression (S1 Fig). Overall, the vaccine showed potentially favorable biophysical and biochemical attributes, indicating that it deserves further development.

### 3. Prediction of secondary and tertiary structure

**Fig 2:**
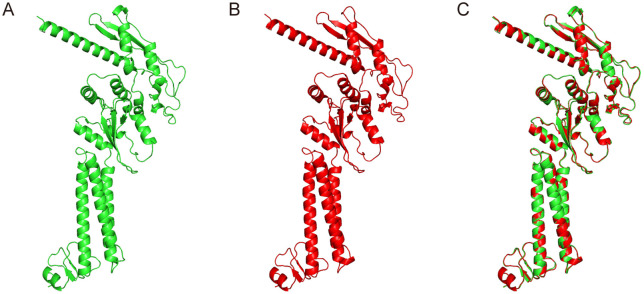
Refined tertiary structure of BtHKU5-CoV-2 vaccine construct. **(A)** Original tertiary structure model of the vaccine construct. **(B)** Refined tertiary structure model of the vaccine construct. **(C)** Structural alignment of the original and refined models, generated and analyzed using PyMOL.

**Fig 3:**
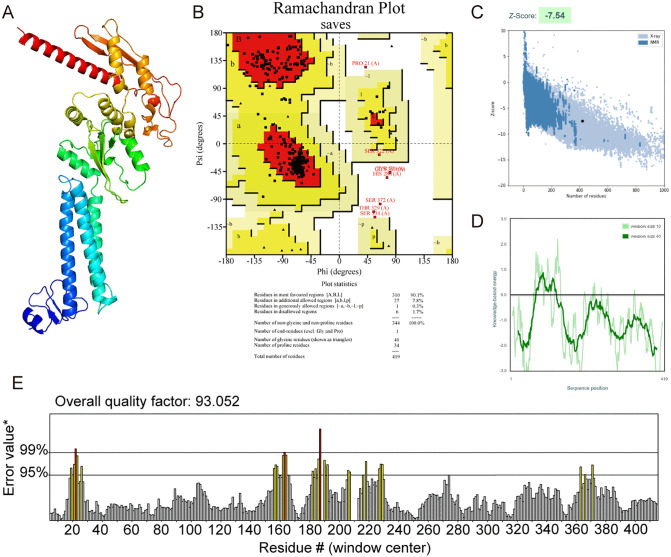
Quality evaluation for refined tertiary structure of BtHKU5-CoV-2 vaccine. **(A)** Refined tertiary structure model of the vaccine. **(B)** The stereochemical quality of the refined tertiary structure was assessed using a Ramachandran plot. In the presented results, red represent the most favoured regions, yellow represent allowed regions and white represent the disallowed regions. Square and triangular dots represent residues distributed in different regions of the plot. **(C)** Evaluation of the Z-score of vaccine’s refined tertiary structure was performed by ProSA-web server. **(D)** Energy map of the refined structure. Most regions of the structure show negative energy values. **(E)** In the ERRAT plot, yellow and red represent error values exceeding the 95% and 99% thresholds, respectively. The error values of most regions in the protein were lower than the 95% confidence limit.

The secondary structure of the vaccine comprised of 25.5% extended strands, 31% alpha helix, and 43.4% random coil, as predicted by the PSIPRED server (Fig 1B). The vaccine’s tertiary structure was modeled using the Robetta web tool, which predicts the 3D protein model through Continuous Automated Model EvaluatiOn (CAMEO) (Fig 2A). Subsequently, the three-dimensional structure of the vaccine was submitted to GalaxyRefine to refine the side chains, and a refined model with a MolProbity score of 2.006 was obtained (Fig 2B). The superimposition result indicated that only the terminal regions of the original structure were needed to be refined to reduce steric clashes (Fig 2C). The quality of the refined construct was evaluated using a Ramachandran plot, and the results showed that 90.1% of residues were in the favored region and 8.1% in the allowed region, suggesting that the overall conformation was predicted to be considered reasonable (Fig 3A-3B). Moreover, ProSA-web analysis showed that the Z-score of the tertiary structure was -7.54, which falls within the range typically observed for native proteins of similar size determined by X-ray crystallography. This suggested that the refined construct may have acceptable structural quality (Fig 3C). The energy map showed that the energy values of most residues were less than zero, supporting the structural stability of the refined model (Fig 3D). According to the ERRAT analysis, the error values of most regions in the protein were below the 95% confidence limit, and the overall quality score was 93.052, which suggests an acceptable model quality (Fig 3E). Together, these *in silico* analyses suggested that the refined tertiary structure of the vaccine exhibited structural plausibility.

### 4. Prediction of conformational B cell epitopes

**Fig 4:**
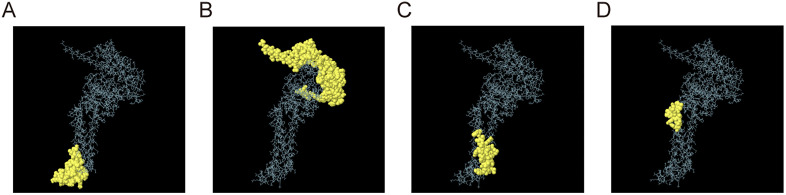
The conformational B cell epitopes within BtHKU5-CoV-2 vaccine tertiary structure. **(A-D)** The conformational B cell epitopes predicted to be presented on the surface model are colored in yellow. These epitopes are distributed at different positions on the vaccine’s tertiary structure.

To identify conformational B-cell epitopes located in the vaccine’s tertiary structure, the 3D model was submitted to the ElliPro web server. The analysis showed four conformational B-cell epitopes predicted to be located on the surface of the tertiary structure, with scores ranging from 0.56 to 0.821 (Table 4). The locations of these epitopes in the three-dimensional structure were shown in Fig 4A-4D. These findings suggested that the vaccine construct possesses potential antibody recognition sites capable of inducing B cell-mediated humoral immune responses.

**Table 4:** Conformation B epitope screening results.

No.	Residues	Number of residues	Score
1	A:G1, A:I2, A:G3, A:D4, A:P5, A:V6, A:T7, A:C8, A:L9, A:K10, A:S11, A:G12, A:A13, A:I14, A:C15, A:H16, A:P17, A:V18, A:F19, A:C20, A:P21, A:R23, A:Y24, A:K25, A:Q26, A:I27, A:G28, A:T29, A:C30, A:G31, A:L32, A:P33, A:G34, A:T35, A:K36, A:C37, A:C38, A:K39, A:K40, A:P41, A:E42, A:A43, A:A44, A:A45, A:K46, A:M47, A:E48, A:A49, A:A50, A:Y51, A:T52, A:A53	52	0.821
2	A:K226, A:G227, A:P228, A:G229, A:P230, A:G231, A:Q265, A:G266, A:P267, A:G268, A:P269, A:A290, A:E291, A:A292, A:L293, A:E294, A:R295, A:A296, A:G297, A:S298, A:D299, A:G300, A:Q301, A:S302, A:A303, A:S304, A:M305, A:T306, A:E307, A:R308, A:Q310, A:M311, A:K314, A:F316, A:P317, A:L318, A:S319, A:A321, A:S322, A:Y323, A:L324, A:R325, A:P326, A:G327, A:S328, A:T329, A:G330, A:P331, A:K332, A:K333, A:S334, A:M335, A:T336, A:E337, A:R338, A:V339, A:Q340, A:M341, A:T342, A:F343, A:V344, A:S346, A:T348, A:K350, A:K351, A:D352, A:V353, A:E354, A:S355, A:G356, A:V357, A:Y358, A:S359, A:V360, A:S361, A:S362, A:F363, A:E364, A:A365, A:K366, A:S367, A:K368, A:K369, A:P370, A:G371, A:S372, A:T373, A:G374, A:P375, A:T376, A:A377, A:E378, A:F379, A:N380, A:Y381, A:R382, A:Q383, A:D384, A:F385, A:W397, A:T398, A:L399, A:K400, A:A401, A:A402, A:A403, A:E404, A:A405, A:A406, A:A407, A:K408, A:T409, A:G410, A:A411, A:L412, A:L413, A:A414, A:A415, A:G416, A:A417, A:A418, A:A419	122	0.709
3	A:R22, A:F89, A:A92, A:A93, A:K95, A:T96, A:Y97, A:S98, A:N99, A:I100, A:T101, A:I102, A:A103, A:A104, A:A105, A:Y106, A:T107, A:Y108, A:S109, A:N110, A:I111, A:T112, A:I113, A:A114, A:M115, A:A116, A:A117, A:Y118, A:M121	29	0.708
4	A:F74, A:H75, A:N76, A:I77, A:N78, A:Y79, A:A81, A:Y82, A:T85, A:N86	10	0.56

### 5. Molecular docking

**Fig 5:**
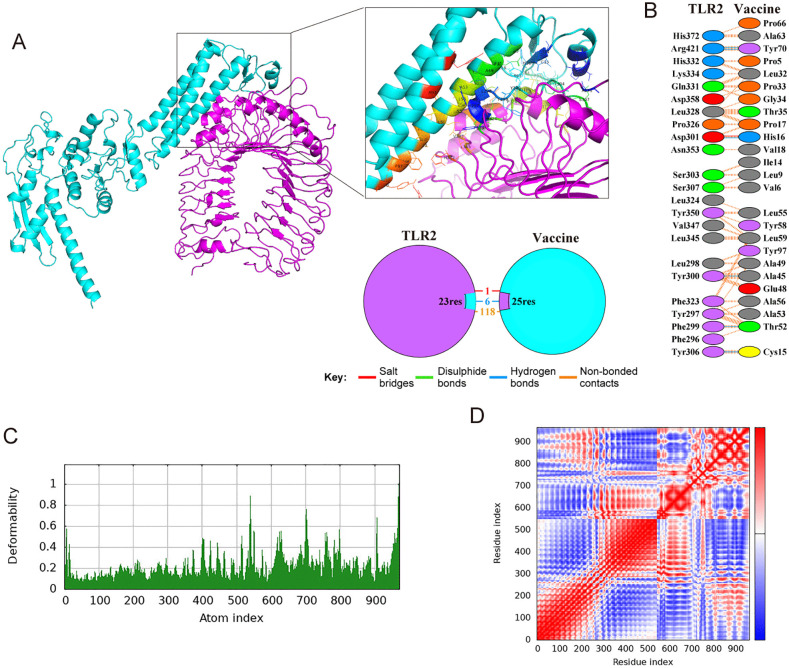
Docking studies of BtHKU5-CoV-2 vaccine-TLR2 complex. **(A)** Docking complex of BtHKU5-CoV-2 vaccine and TLR2. The vaccine is shown in light turquoise, while TLR2 is shown in purple. The box highlights the interacting residues between the two molecules. A simplified diagram below the box depicts the various forces and bonds between the vaccine and TLR2. **(B)** The connections, including salt bridges, disulphide bonds, hydrogen bonds and non-bonded contacts, predicted between amino acids in the docking complex, are represented in red, yellow, blue and orange, respectively. **(C)** Deformability plot of the complex. The graph describes the flexibility of each atom in the modeled structure. **(D)** Covariance plot of the complex. The plot illustrates the correlated and anti-correlated motions among residues. Red, white and blue colors indicate correlated motion, non-correlated motion, anti-correlated motion, respectively.

**Fig 6:**
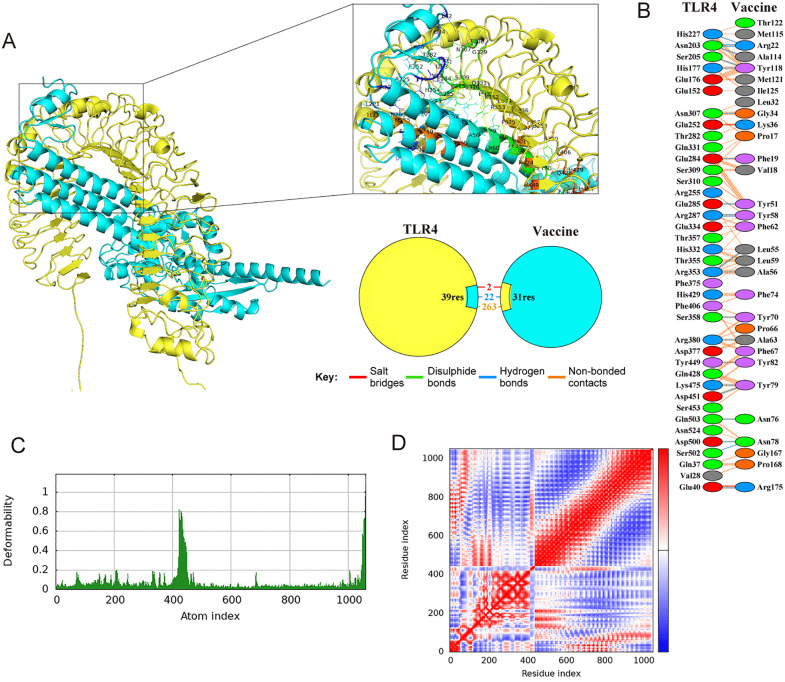
Docking studies of BtHKU5-CoV-2 vaccine-TLR4 complex. **(A)** Docking complex of BtHKU5-CoV-2 vaccine and TLR4. The vaccine is shown in light turquoise, while TLR4 is shown in yellow. The box highlights the interacting residues between the two molecules. A simplified diagram below the box depicts the various forces and bonds between the vaccine and TLR4. **(B)** The connections, including salt bridges, disulphide bonds, hydrogen bonds and non-bonded contacts, predicted between amino acids in the docking complex, are represented in red, yellow, blue and orange, respectively. **(C)** Deformability plot of the complex. The graph describes the flexibility of each atom in the modeled structure. **(D)** Covariance plot of the complex. The plot illustrates the correlated and anti-correlated motions among residues. Red, white and blue colors indicate correlated motion, non-correlated motion, anti-correlated motion, respectively.

TLR2 and TLR4 are critical receptors for recognizing viral glycoproteins, thereby activating the immune response [30]. Therefore, molecular docking between the vaccine and these proteins was performed using ClusPro 2.0. We selected the docking model based on the following criteria: higher cluster rank, lower energy-weighted score, and reasonable binding mode (Table 5). The docking complexes were then refined using the HADDOCK 2.4 server (Figs 5A and 6A). The preferred model was selected based on the HADDOCK score, van der Waals energy, electrostatic energy, and desolvation energy (Table 5). To explore the interaction between the vaccine and the indicated proteins, the PDBsum server was used to analyze the docking complexes (Table 5). Our results suggested that the vaccine was predicted to form 6 hydrogen bonds, 1 salt bridge, and 118 non-bonded contacts with TLR2 (Fig 5A-5B), while it was predicted to form 22 hydrogen bonds, 2 salt bridges, and 263 non-bonded contacts with TLR4 (Fig 6A-6B). In addition, deformability analysis suggested limited predicted flexibility for both the TLR2-vaccine and the TLR4-vaccine complexes (Figs 5C and 6C). The covariance plot also indicated that both complexes had reasonable dynamic coupling relationships between internal residues (Figs 5D and 6D). These *in silico* results suggested the structural plausibility of potential interactions between the vaccine construct and TLR2 and TLR4. Nonetheless, experimental validation, such as TLR reporter assays and cytokine profiling, is required to determine whether the vaccine can activate TLR2 and TLR4 receptor.

**Table 5:** Results of molecular docking.

Complex	Vaccine-TLR2	Vaccine-TLR4
	ClusPro 2.0	
Center Weighted Score	-1413.6	-1602
Lowest Energy Weighted Score	-1693.2	-1768.1
	HADDOCK 2.4	
HADDOCK score	-152.8 + /- 1.2	-132.9 + /- 16.0
RMSD from the overall lowest-energy structure	6.2 + /- 0.5	4.3 + /- 0.4
Van der Waals energy	-87.4 + /- 5.6	-75.0 + /- 9.1
Electrostatic energy	-159.1 + /-17.9	-261.7 + /- 75.0
Desolvation energy	-57.1 + /- 2.1	-38.2 + /- 8.9
Buried Surface Area	3030.4 + /- 76.1	3119.0 + /- 193.5
Z-Score	-1	-1.5
	PDBsum	
Number of hydrogen bonds	6	22
Number of salt bridges	1	2

### 6. Population coverage calculation

**Fig 7:**
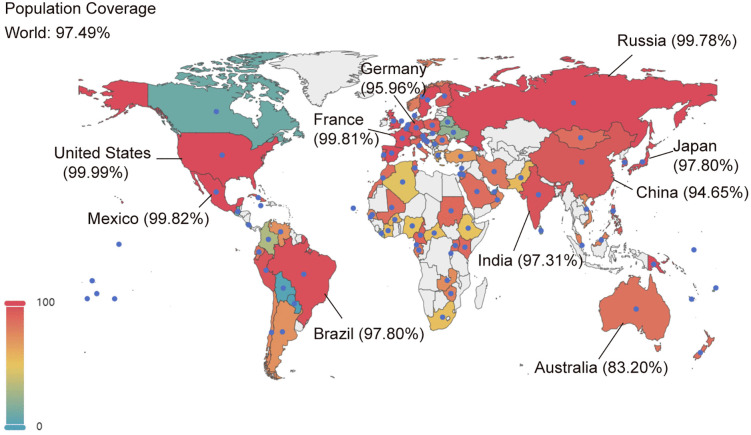
Population coverage of BtHKU5-CoV-2 vaccine across the world. This map depicts the proportion of the population covered by the designed vaccine in various countries and regions. Countries and regions that have available data are labeled with blue dots. The global population coverage was visualized with pyecharts, utilizing a base map layer from Natural Earth (Natural Earth, 1:110 million Cultural Vectors, available at https://www.naturalearthdata.com/downloads/110m-cultural-vectors/; data publicly available at https://www.naturalearthdata.com/ under the Public Domain license).

To estimate the percentage of individuals who may be able to present the selected epitopes across the world, the vaccine’s population coverage was calculated using the IEDB database. As shown in Fig 7, approximately 97.49% of the global population was predicted to be covered. Most regions show predominantly high population coverage (e.g., Europe 99.76%, North America 99.96%), with only a few regions exhibiting lower coverage (e.g., South Africa 51.86%, Central America 23.88%) (S2 Table). In particular, the vaccine was predicted to achieve coverage rates of 99.99% in the United States, 94.65% in China, 99.81% in France, and 99.78% in Russia (Fig 7). Overall, the prediction suggests that the designed vaccine may achieve broad HLA-based population coverage globally.

### 7. *In silico* immunization simulation

**Fig 8:**
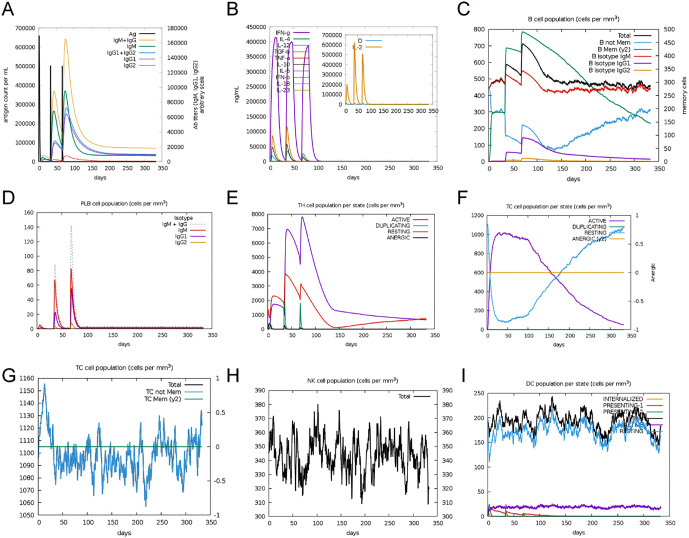
Immunological simulation analysis following administration of three doses of the BtHKU5-CoV-2 vaccine. The antibody titers (IgM, IgG, and IgG subclasses) **(A)** and the levels of cytokines (IFN-γ, TGF-β, and IL-10) **(B)** were increased following three-dose vaccine administration. **(C)** The number of total B cells, memory B cells, and isotype-specific B cells increased after vaccination. **(D)** Plasma B-cell population (PLB), including IgM + IgG, IgM, and IgG1 isotype, exhibited transient expansion. **(E)** The population of activated Th cells surged following vaccine injection. **(F)** The active Tc cells expanded rapidly after immunization, and then the T cell population gradually shifted to a predominantly resting state. The populations of effector Tc cells **(G)**, total NK cells **(H)**, and DCs in presenting or resting states **(I)** were maintained at a stable level throughout the simulation period.

To explore the potential dynamic immune response *in silico* following vaccination, the C-IMMSIM server was employed to predict the immune response profile of the mRNA vaccine. This server simulated the immunogenic profile following three vaccine injections for a period of approximately one year. As shown in Fig 8A, the antibody levels were increased following administration of three vaccine doses, with the level of IgM + IgG peaking at approximately 1.6 × 10^5^, suggesting enhanced humoral responses in the simulation. Although IFN-γ and IL-2 were predicted to increase once the vaccine was administered, they were quickly cleared from the plasma (Fig 8B). During the simulation period, the populations of B cells, plasma cells, and helper T (Th) cells initially were predicted to expand following injection, while numerous resting cytotoxic T (Tc) cells were activated and transformed into active Tc cells (Fig 8C-8F). These results suggest that the vaccine may have the potential to activate the host’s cellular immune response. In addition, the total populations of Tc cells, natural killer (NK) cells, and dendritic cells (DCs) consistently increased following administration of the first dose of the vaccine, and eventually remained in equilibrium in a dynamic range, suggesting relatively stable innate immune dynamics within the simulation (Fig 8G-8I). Therefore, these computational simulation results suggest that the mRNA vaccine designed in this study may have the potential to elicit a robust and sustained adaptive immune response.

### 8. mRNA vaccine construction

After attaching the tPA and MITD sequences to the N-terminus and C-terminus of the vaccine, respectively, the full-length amino acid sequence of the final mRNA vaccine was submitted to the GenSmart Codon Optimization tool for reverse translation and codon optimization. The improved cDNA fragment showed a Codon Adaptation Index (CAI) value of 0.91 and a 61% GC content, suggesting a high predicted expression potential in human cells (S2 Fig). Upon merging the 5’-UTR, 3’-UTR, and poly(A) tail, the secondary structure of the full-length RNA sequence was analyzed using an RNAfold server (S3 Fig). The free energy of the thermodynamic ensemble was predicted to be -678.61 kcal/mol, suggesting a stable RNA structure. Overall, the mRNA construct shows a stable predicted RNA secondary structure and exhibits a high CAI value in the coding sequence, implying a potentially long intracellular half-life and efficient expression in the host. The encoded protein displays high predicted solubility, supporting its low aggregation propensity and suitability for *in vivo* expression.

**Fig 9:**
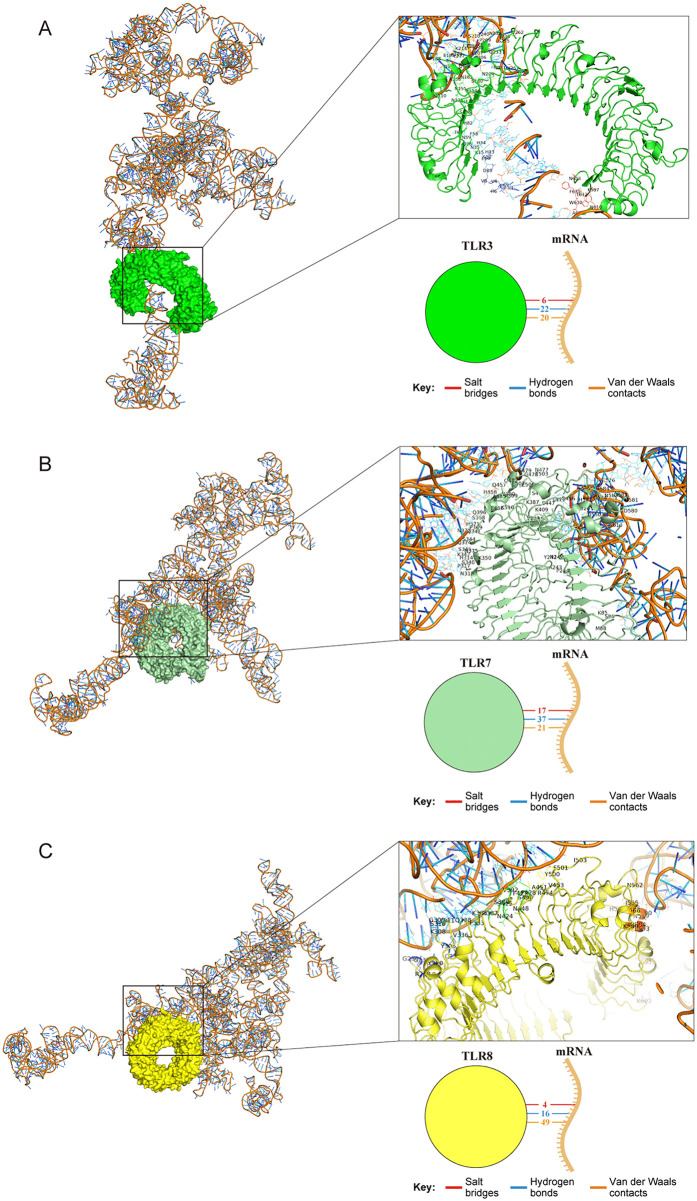
Docking analysis of BtHKU5-CoV-2 vaccine (mRNA)-TLR complexes. **(A)** Docking models of the vaccine mRNA with TLR3. The box highlights the interacting residues between the mRNA and TLR3. The simplified diagram below the box illustrates the various forces and bonds involved. **(B)** Docking models of the vaccine mRNA with TLR7. The interacting residues between the mRNA and TLR7 are shown in the box. The corresponding interaction forces and bonds are summarized in the schematic below. **(C)** Docking models of the vaccine mRNA with TLR8. The boxed region shows the interacting residues between the mRNA and TLR8, with the simplified diagram below depicting the various forces and bonds.

TLR3, TLR7, and TLR8 are critical sensors of exogenous nucleic acids and are located in endosomes [35]. We first predicted the binding between the vaccine’s full-length mRNA and these TLRs using the RPISeq tool based on RF and SVM methods. All binding scores exceeded 0.5, suggesting that the vaccine mRNA may interact with TLR3, TLR7, and TLR8 (S3A Table). We then ran a molecular docking analysis for TLR3, TLR7, and TLR8 using the HDOCK server. The docking models were shown in Fig 9A-9C. The *in silico* results predicted that the mRNA may form potential contacts with TLRs through hydrogen bonds, salt bridges, and van der Waals contacts. The binding and confidence scores were presented in S3B Table. All binding scores were less than -200 and all confidence scores exceeded 0.9, indicating that the vaccine mRNA may have the potential to interact with TLR3, TLR7, and TLR8.

Finally, the full-length DNA of the mRNA vaccine was introduced into the pET-28a (+) vector via homologous recombination (S4 Fig). The plasmid was used as a template for *in vitro* transcription, and the synthesized mRNA was encapsulated by lipid nanoparticles and used for vaccination.

## Discussion

Since the first discovery of BtHKU5-CoV-2 in March 2025, widespread concerns have been raised [5–7]. BtHKU5-CoV-2 was found to infect human cell lines by utilizing the human ACE2 receptor, similar to SARS-CoV-1 and SARS-CoV-2, which led to severe acute respiratory syndrome (SARS) and the COVID-19 pandemic, respectively [1,36,37]. Moreover, it adapts to ACE2 orthologs of many mammalian and avian species, indicating that it might have a wide host range [1]. Although no case of human infection has been reported, BtHKU5-CoV-2 has the potential to spill over to humans and cause a pandemic outbreak. The team of Prof. Zhengli Shi demonstrated that some protease inhibitors (such as E64d), endosomal acidification inhibitors (such as bafilomycin A1), one fusion inhibitor EK1C4, and several small molecule inhibitors (including nirmatrelvir and remdesivir) suppressed the entry and replication of BtHKU5-CoV-2 *in vitro* [1]. Nevertheless, it is necessary to develop prophylactic and therapeutic vaccines against BtHKU5-CoV-2. Engagement of host receptor recognition and viral and cell membrane fusion is mediated by spike glycoprotein on the surface of coronaviruses [38]. Therefore, the spike glycoprotein is a critical target for the development of prophylactic vaccines. During the response to outbreaks or rapidly evolving pathogens, nucleic acid vaccines present more advantages than traditional vaccines, including inactivated vaccines and subunit vaccines [39]. The success of combating COVID-19 has accelerated the development of mRNA vaccines.

In the present study, we designed a multi-epitope mRNA vaccine candidate for BtHKU5-CoV-2. The epitope sequences were obtained from the conserved regions of the spike glycoprotein of BtHKU5-CoV-2, ensuring that the multi-epitope vaccine could cover all six BtHKU5-CoV-2 strains. Eight CTL epitopes, seven HTL epitopes, and five LBL epitopes with favorable predicted antigenicity and immunogenicity were selected for vaccine construction. These epitopes were expected to trigger antibody-mediated immunity and T-cell immune responses. In addition, the selected epitopes showed no significant sequence homology to the human proteome or to proteins from human gut commensal bacteria based on our predefined screening criteria. Such homology filtering can reduce the theoretical risk of off-target effects and cross-reactivity. However, autoimmune potential and overall safety of the vaccine construct still require experimental assessment. In accordance with previous studies, linkers AAY, GPGPG, KK, and EAAAK were used to connect the indicated epitopes [40,41]. Incorporating linkers was expected to facilitate accurate cleavage and proper folding of the multi-epitope vaccine. β-defensin II acts as a ligand for TLR receptors, thereby activating the immune response [42,43]. Thus, β-defensin II was attached to the N-terminus of the vaccine construct and acted as an adjuvant. Furthermore, a cell entry peptide, the TAT sequence, was added to the C-terminus of the vaccine construct, and it was proposed to target vaccines across the cellular membrane [44].

To further determine whether the protein encoded by the mRNA vaccine adopted a proper folding conformation, we predicted the tertiary structure using the Robetta server. The stereochemical quality of the refined tertiary structure was assessed using a Ramachandran plot, and the results indicated that the model possessed good spatial structural quality. A comprehensive understanding of the interaction between vaccines and immune receptors, especially TLR2 and TLR4, is crucial for vaccine development. Molecular docking analysis suggested that the β-defensin II domain of the vaccine exhibited favorable docking poses with TLR2 and TLR4. In addition, hydrogen-bond and salt-bridge analyses support the structural plausibility of potential interactions between the refined vaccine construct and TLR2 and TLR4. Nevertheless, these findings are hypothesis-generating, and docking analysis alone cannot establish physical binding, receptor activation, or downstream signaling. Functional assays, such as TLR pathway reporter assays and cytokine profiling, are required to determine whether the vaccine construct is capable of activating TLR2 and TLR4.

Previous studies have demonstrated that the RNA of SARS-CoV-2 is recognized by TLR3, TLR7, and TLR8, resulting in the activation of innate immune signaling and production of cytokines and interferons [45,46]. The RNA-protein interaction prediction scores obtained in this study suggested that the mRNA of the designed vaccine may exhibit favorable predicted binding propensity toward TLR3, TLR7, and TLR8. Together, the vaccine mRNA may assist in triggering the host immune response.

The immune responses induced by the developed vaccine was predicted using the C-IMMSIM server. In the simulation, the levels of antibodies were increased after the vaccine injection. In addition, vaccination was predicted to increase the number of Th cells and active Tc cells in the peripheral blood, suggesting the potential for cellular immune activation. Furthermore, the vaccine was predicted to induce long-lasting immunity *in silico*, as the antibodies, including IgG and IgM, were predicted to remain stable for a long period of time, and memory B cells and memory T cells were predicted to be generated after administration of three vaccines doses. Collectively, the results of our study suggest that the designed mRNA vaccine has the potential to induce both humoral and cellular immune responses.

Although the predicted global population coverage of the vaccine construct was 97.49%, coverage varied substantially across regions, with notably lower estimates in South Africa (51.86%) and Central America (23.88%). Population coverage depends on several factors: (i) a given epitope can trigger T-cell responses only in individuals who express HLA alleles capable of binding and presenting the epitope; (ii) HLA molecules are highly polymorphic; and (iii) HLA allele frequencies differ markedly across populations and geographic regions [33]. In addition, the datasets used for population coverage estimates may vary in sample size and representativeness across geographic groups. Therefore, low predicted coverage in certain regions may indicate that a considerable proportion of individuals are unable to present the selected epitopes, potentially limiting real-world reach. Further optimization could be carried out to incorporate additional epitopes based on local HLA distributions, which could improve the coverage in underrepresented populations. However, experimental validation is required to confirm epitope presentation and immunogenicity of the vaccine construct in individuals with diverse HLA backgrounds.

The stepwise experimental validation roadmap could include: (i) confirmation of epitope presentation using HLA immunopeptidomics; (ii) assessment of TLR receptor activation by TLR reporter assays and cytokine profiling; (iii) evaluation of vaccine immunogenicity in appropriate animal models (e.g., HLA-transgenic mice) following mRNA vaccination, including measuring epitope-specific IgG titers by ELISA and determining the percentage of CD4 + IFN-γ + T cells and CD8 + IFN-γ + T cells in PBMCs by flow cytometry; and (iv) protective efficacy could be evaluated in challenge studies, in which vaccinated mice are challenged with BtHKU5-CoV-2.

In the digital era, immunoinformatics is increasingly playing critical roles in public health strategies, including enhancing epidemic preparedness and accelerating vaccine development. Immunoinformatics analyses can be employed to evaluate the cross-reactive potential of existing vaccines against newly emerging pathogens by mapping conserved epitopes. In addition, this approach minimizes reliance on conventional screening, lowers development costs, and facilitates faster clinical translation [47–49]. Moreover, by leveraging *in silico* prediction tools, vaccinologists can accurately identify immunogenic epitopes, thereby shortening the development timeline for mRNA vaccines [48]. Previous studies have revealed that over 70% of emerging infectious diseases originate from animal reservoirs. Therefore, it is important to proactively establish a vaccine bank against zoonotic pathogens [49,50], for which mRNA vaccines represent an appropriate option. Given that BtHKU5-CoV-2 has the potential for spillover and may lead to an epidemic, it is necessary to develop an mRNA vaccine against BtHKU5-CoV-2.

### The limitations of this study

Although numerous *in silico* prediction tools have been progressively refined, some limitations are inherent in their algorithms. For example, many epitope predictors possess restricted capabilities of fully identifying appropriate antigen-processing sites, which means that these models remain insufficient to recapitulate the complexity of the immune response *in vivo* [51]. In addition, relying on only a small set of selected tools may lead to systematic bias in the results. For instance, NetMHCpan may show low accuracy for underrepresented alleles because it is typically trained and validated on a limited set of representative MHC alleles [52]. Moreover, different tools might yield discordant outputs on the same dataset [52]. Cross-tool validation also has inherent limitations. Consequently, *in silico* prediction cannot fully substitute for experimental assays.

Although the immunoinformatics pipeline employed in this study can substantially accelerate candidate epitope prioritization and vaccine design by reducing time and cost, the *in silico* outputs should be interpreted as hypothesis-generating predictions that support candidate ranking, rather than as evidence of confirmed immune induction, protection, or other biological processes. A key limitation of the present work is the lack of experimental validation. Therefore, future work should include experiments such as assays to confirm epitope presentation, receptor binding and functional activation, as well as *in vivo* evaluation of immunogenicity and protective efficacy.

Despite the rapid development of mRNA vaccine technology, several critical challenges still remain to be addressed. First, substantial efforts are needed to enhance the intracellular stability of mRNA, for example, by optimizing the 5′ cap structure, untranslated regions (UTRs), and the poly(A) tail [53]. Although lipid nanoparticles (LNPs) are the most clinically advanced delivery system for mRNA vaccines, targeted delivery to specific immune cell subsets remains limited and warrants further improvement. In addition, PEGylated components in some LNP formulations have been associated with allergic reactions in a few people vaccinated with SARS-CoV-2 vaccine [53,54]. With widespread deployment of SARS-CoV-2 mRNA vaccines, several adverse events have been reported, such as blood coagulation dysfunction, autoimmune hepatitis, and myocarditis. Although the overall incidence is low, further optimization of delivery vehicles and vaccine adjuvants of mRNA vaccines remains necessary to improve the safety profile [55]. Despite these limitations, mRNA vaccines remain an extraordinary tool for rapid response to emerging pandemics and holds promise for developing countermeasures against potential zoonotic viruses, such as BtHKU5-CoV-2.

## Supporting information

S1 Fig Vaccine solubility was predicted using the Protein-Sol tool. QuerySol denotes the solubility of the vaccine, and PopAvrSol represents the average solubility of proteins in the experimental dataset.
(TIF)

S2 Fig Codon adaptation index (CAI) analysis of the coding regions of BtHKU5-CoV-2 vaccine. The plot shows the relative usage frequency of codons by the host. The relative frequency of the majority of codons exceeds 70%. The CAI and GC content of the vaccine are 0.91 and 61%, respectively.
(TIF)

S3 Fig Secondary structure of the mRNA sequence of the BtHKU5-CoV-2 vaccine. (A) The MFE secondary structure predicted at minimum free energy prediction. (B) The centroid secondary structure representing the thermodynamic ensemble prediction.
(TIF)

S4 Fig Schematic diagram of the BtHKU5-CoV-2 mRNA vaccine plasmid. The inserted sequence is the full-length DNA encoding the BtHKU5-CoV-2 vaccine. A *BamHI* restriction site was used for plasmid linearization.
(TIF)

S1 Table Immunoinformatics tools, parameters, and cutoff values used for epitope prediction and vaccine construct design. (DOCX)

S2 Table Population coverage of BtHKU5-CoV-2 mRNA vaccine across different geographical regions. (DOCX)

S3 Table Prediction of RNA-protein interaction for the BtHKU5-CoV-2 mRNA vaccine using different tools. (A) RNA-protein interaction prediction by RPISeq tool. (B) RNA-protein interaction prediction by HDOCK server.
(DOCX)
