# Supplementary material for: In silico design of a novel multi-epitope mRNA vaccine candidate for BtHKU5-CoV-2 using immunoinformatics
Source: PLoS Negl Trop Dis. 2026 Apr 3;20(4):e0013517. doi: 10.1371/journal.pntd.0013517 (PMC13065043; doi:10.1371/journal.pntd.0013517)
Supplement: S1 Table — (DOCX) [file pntd.0013517.s005.docx]

S1 Table. Immunoinformatics tools, parameters, and cutoff values used for epitope prediction and vaccine construct design.

| **Tool** | **Parameter(s)** | **Threshold(s)** | **Cutoff Justification** |
| --- | --- | --- | --- |
| NetCTL 1.2 | 1. Weight on cleavage = 0.15; TAP = 0.05  2. HLA supertypes: A1, A2, A3, A24, A26, B7, B8, B27, B39, B44, B58, and B62. | Epitope identification score > 0.75 | Default threshold recommended by developers to maximize sensitivity and specificity [1]. |
| TepiTool | 1. Class I HLA alleles: panel of 27 most frequent A & B alleles.  2. Conservancy analysis: No  3. Prediction method: NetMHCpan (v4.1).  4. Select peptides based on predicted lC50. | IC50 < 500 nM | The IEDB recommends an absolute binding affinity (IC50) threshold of 500 nM identifies binders. |
| NetMHCIIpan - 4.0 | 1. Peptide length: 15 mer  2. HLA: HLA alleles indicated in Methods section. | IC50 < 500 nM (binder) | A 500 nM affinity threshold is commonly used in previous NetMHCIIpan studies [2] and is applied here for comparative purposes. For EL scores, %Rank < 10 is typically considered indicative of binding. |
| IEDB Class I Immunogenicity | Specify which positions to mask: Default (1st, 2nd, and C-terminus amino acids) | Score > 0 | Peptides with positive scores are predicted to have higher immunogenic potential [3]. (Used for ranking only; not definitive evidence of immunogenicity.) |
| ABCpred tool | 1. Window length to use for prediction: 16  2. Overlapping filter: ON | Score ≥ 0.51 | 1. The default threshold is 0.51.  2. At 0.5, it shows 65.93% accuracy with equal sensitivity and specificity using window length of 16 [4]. |
| Vaxijen 2.0 | Target organism: Virus | Score > 0.4 | It shows 70% accuracy at threshold 0.4 [5]. |
| AllerTOP 2.0 | N/A (no adjustable parameter) | No user-defined threshold. | Fixed binary output (probable allergen / non-allergen). |
| ToxinPred 2.0 | 1. Machine Learning Technique used for developing model: Hybrid (RF+BLAST+MERCI)  2. Threshold value: 0.6 | Hybrid Score ≤ 0.6 | 1. The default threshold is 0.6.  2. The default threshold of is commonly used and reported in reviews [6]. |
| IL2Pred | 1. Job type: Il2 versus Il2 non-inducer  2. Machine Learning Technique used for developing model: Hybrid (ET + MERCI)  3. Threshold value: 0.5 | Hybrid Score ≥ 0.5 | The default threshold is 0.5 [7]. Epitopes were marked “+” when the Hybrid Score ≥ 0.5 and “−” otherwise. (Used for prioritization only; not evidence of cytokine induction.) |
| IL4Pred | 1. Model for prediction: Hybrid (SVM + motif) based  2. SVM threshold: 0.2 | SVM Score ≥0.2 | At 0.2, it shows maximum accuracy of 75.76% [8]. Epitopes were assigned “+” when the SVM score ≥ 0.2. (Used for prioritization only; not evidence of cytokine induction.) |
| IFNepitope | 1. Approach for predicting IFN-gamma epitopes: Motif and SVM hybrid  2. Model for prediction: IFN-gamma versus Non IFN-gamma | No numerical cutoff required. | Binary output: “Positive” (IFN-γ–inducing) or “Negative” (non-inducing). “+” was assigned to epitopes classified as “Positive”. (Used for prioritization only; not evidence of cytokine induction.) |
| ProtParam tool | Input: amino acid sequence only | No user-defined thresholds required. |  |
| PSIPRED 4.0 | Input: amino acid sequence only | No user-defined thresholds required. |  |
| Robetta service | Modeling method: RoseTTAFold | No user-defined thresholds required. |  |
| GalaxyRefine server | 1. Services: RefineComplex  2. Symmetric refinement  for homo-oligomer: No | No user-defined thresholds required. |  |
| ProSA-Web | Upload a structure in PDB format only. | No user-defined thresholds required. |  |
| PROCHECK | Upload PDB-format file only. | No user-defined thresholds required. |  |
| ClusPro 2.0 server | Upload PDB-format files of receptor and ligand only. | No user-defined thresholds required. |  |
| HADDOCK 2.4 server | 1. Upload PDB-format files of protein and ligand.  2. Label the active residues directly involved in the interaction. | No user-defined thresholds required. |  |
| PDBsum server | Upload PDB-format file docking complex only. | No user-defined thresholds required. |  |
| iMODs tool | 1. Mode: basic  2. Coarse-grained model: C5 | No user-defined thresholds required. |  |
| ERRAT | Upload a structure in PDB format only. | No user-defined thresholds required. |  |
| ElliPro | 1. Minimum score: 0.5 (Default)  2. Maximum distance (Angstrom): 6 (Default) | Score ≥ 0.5 | ElliPro achieved the best performance (AUC values) with S = 0.5 and R = 6 Å when average or significance-based predictions were considered [9]. |
| Population Coverage server | 1. Query by area country ethnicity.  2. Calculation option(s): Class I and II combined.  3. Area(s) and/or population(s): select all.  4. Enter epitope / MHC restriction data: The input includes the vaccine-derived epitopes and their respective HLA restrictions. | No user-defined thresholds required. |  |
| C-IMMSIM webserver | 1. Random Seed: 12345 (default)  2. Simulation Volume:  10 (default)  3. Simulation Steps:  1000  4. Host HLA selection: HLA-A0201, HLA-A2301, HLA-B5301, HLA-B1501, HLA-DRB1_0405, and HLA-DRB1_0701  5. Number of injections: 3  6. What to inject: vaccine (no LPS)  7. Time step of injection: 1, 100, 200  8. Adjuvant: 100 (default)  9. Num Ag to inject: 1000 (default) | No user-defined thresholds required. |  |
| RNAfold tool | 1. Fold algorithms and basic options: minimum free energy (MFE) and partition function; avoid isolated base pairs.  2. Dangling end options: dangling energies on both sides of a helix in any case (default).  3. Energy Parameters: RNA parameters (Turner model, 2004)  4. Rescale energy parameters to given temperature: 37℃ (default).  5. Use this salt concentration in molar (M): 1.021 (default) | No user-defined thresholds required. |  |
| RPISeq tool | Upload a single RNA and multiple protein sequences only. | No user-defined thresholds required. |  |
| GenSmart Codon Optimization tool | 1. Upload Gene sequence.  2. Expression Host Organism: Human | No user-defined thresholds required. |  |

**Reference:**

1. Larsen MV, Lundegaard C, Lamberth K, Buus S, Lund O, Nielsen M. Large-scale validation of methods for cytotoxic T-lymphocyte epitope prediction. BMC Bioinformatics. 2007;8:424.

2. Jensen KK, Andreatta M, Marcatili P, Buus S, Greenbaum JA, Yan Z, et al. Improved methods for predicting peptide binding affinity to MHC class II molecules. Immunology. 2018;154(3):394-406.

3. Calis JJ, Maybeno M, Greenbaum JA, Weiskopf D, De Silva AD, Sette A, et al. Properties of MHC class I presented peptides that enhance immunogenicity. PLoS Comput Biol. 2013;9(10):e1003266.

4. Saha S, Raghava GP. Prediction of continuous B-cell epitopes in an antigen using recurrent neural network. Proteins. 2006;65(1):40-8.

5. Doytchinova IA, Flower DR. VaxiJen: a server for prediction of protective antigens, tumour antigens and subunit vaccines. BMC Bioinformatics. 2007;8:4.

6. Wei Y, Qiu T, Ai Y, Zhang Y, Xie J, Zhang D, et al. Advances of computational methods enhance the development of multi-epitope vaccines. Brief Bioinform. 2024;26(1).

7. Mehta NK, Lathwal A, Kumar R, Kaur D, Raghava GPS. In Silico tool for predicting, designing and scanning IL-2 inducing peptides. Sci Rep. 2025;15(1):25692.

8. Dhanda SK, Gupta S, Vir P, Raghava GP. Prediction of IL4 inducing peptides. Clin Dev Immunol. 2013;2013:263952.

9. Ponomarenko J, Bui HH, Li W, Fusseder N, Bourne PE, Sette A, et al. ElliPro: a new structure-based tool for the prediction of antibody epitopes. BMC Bioinformatics. 2008;9:514.
