# Supplementary material for: In silico design of a novel multi-epitope mRNA vaccine candidate for BtHKU5-CoV-2 using immunoinformatics
Source: PLoS Negl Trop Dis. 2026 Apr 3;20(4):e0013517. doi: 10.1371/journal.pntd.0013517 (PMC13065043; doi:10.1371/journal.pntd.0013517)
Supplement: S2 Table — (DOCX) [file pntd.0013517.s006.docx]

S2 Table. Population coverage of BtHKU5-CoV-2 mRNA vaccine regions across different geographical regions.

| **Continent** | **Region** | **Coverage** |
| --- | --- | --- |
| Asia | East Asia | 95.49% |
|  | South Asia | 97.63% |
|  | Northeast Asia | 94.87% |
|  | Southeast Asia | 87.12% |
|  | Southwest Asia | 78.57% |
| Europe | Europe | 99.76% |
| Oceania | Oceania | 95.58% |
| America | North America | 99.96% |
|  | South America | 89.97% |
|  | Central America | 23.88% |
|  | West Indies | 86.73% |
| Africa | East Africa | 89.28% |
|  | South Africa | 51.86% |
|  | North Africa | 90.94% |
|  | West Africa | 91.54% |
|  | Central Africa | 90.26% |
