# Supplementary material for: In silico design of a novel multi-epitope mRNA vaccine candidate for BtHKU5-CoV-2 using immunoinformatics
Source: PLoS Negl Trop Dis. 2026 Apr 3;20(4):e0013517. doi: 10.1371/journal.pntd.0013517 (PMC13065043; doi:10.1371/journal.pntd.0013517)
Supplement: S3 Table — (A) RNA-protein interaction prediction by RPISeq tool. (B) RNA-protein interaction prediction by HDOCK server. (DOCX) [file pntd.0013517.s007.docx]

S3 Table. RNA-protein interaction of the BtHKU5-CoV-2 mRNA vaccine using different tools.

A. RNA-protein interaction prediction by RPISeq tool

| **Protein** | **RF Classifier** | **SVM Classifier** |
| --- | --- | --- |
| TLR3 | 0.6 | 0.904 |
| TLR7 | 0.65 | 0.807 |
| TLR8 | 0.7 | 0.845 |

B. RNA-protein interaction prediction by HDOCK server

|  | **TLR3** | **TLR7** | **TLR8** |
| --- | --- | --- | --- |
| Docking Score | -416 | -396.21 | -355.84 |
| Confidence Score | 0.9951 | 0.9928 | 0.984 |
